# Detection of Highly Differentiated Genomic Regions Between Lotus (*Nelumbo nucifera* Gaertn.) With Contrasting Plant Architecture and Their Functional Relevance to Plant Architecture

**DOI:** 10.3389/fpls.2018.01219

**Published:** 2018-08-20

**Authors:** Mei Zhao, Ju-Xiang Yang, Tian-Yu Mao, Huan-Huan Zhu, Lin Xiang, Jie Zhang, Long-Qing Chen

**Affiliations:** ^1^Key Laboratory of Horticultural Plant Biology, College of Horticulture and Forestry Sciences, Huazhong Agricultural University, Ministry of Education, Wuhan, China; ^2^Southwest Engineering Technology and Research Center of Landscape Architecture, State Forestry Administration, Southwest Forestry University, Kunming, China

**Keywords:** *Nelumbo nucifera*, genetic resources, plant architecture, whole genome re-sequencing, cell morphology

## Abstract

The lotus (*Nelumbo nucifera* Gaertn.) is one of the most economically and ornamentally important perennial aquatic plants. Plant architecture is an important trait for lotus classification, cultivation, breeding, and applications. In this study, traits representing plant architecture were measured in 390 lotus germplasms for 3 years. According to the phenotypic distribution, 21 large architecture (LA) and 22 small architecture (SA) germplasms exhibiting extreme phenotypes were selected as representatives of plant architecture. Microscopy analyses revealed that LA lotuses possessed far more vertical cells and longer cell lengths than SA lotuses, and there was a closer linear relationship between vertical cell number and plant architecture than cell length and plant architecture. Furthermore, based on whole genome re-sequencing data from 10 LA and 10 SA lotus germplasms, fixation index (F_ST_) genome scan identified 11.02 Mb of genomic regions that were highly differentiated between the LA and SA lotus groups. Chi-square test revealed that 17,154 single nucleotide polymorphisms (SNPs) and 1,554 insertions and deletions (InDels) showed distinct allelic distribution between the LA and SA lotus groups within these regions. A total of 126 variants with distinct allelic distribution in the highly differentiated region were predicted to cause amino acid changes in 60 genes. Among the 41 genes with functional annotation, the expression patterns of six genes involved in cell division and cell wall construction were confirmed using quantitative reverse-transcription PCR (qRT-PCR). In addition, 34 plant architecture-associated InDel markers were developed and verified in the remaining 11 LA and 12 SA lotus plant architecture representatives. This study identified promising functional markers and candidates for molecular breeding and will facilitate further elucidation of the genetic mechanisms underlying plant architecture in the lotus.

## Introduction

Sacred lotus (*Nelumbo nucifera* Gaertn.; 2*n* = 2*x* = 16) is a perennial aquatic plant belonging to the Nelumbonaceae (Bremer et al., 2009). It is a basal eudicot plant with a number of monocot characteristics, and is important in evolutionary and taxonomic studies (Xue et al., 2012). Sacred lotus is extensively distributed throughout Asia and Northern Australia, and plays a vital role in cultural and religious activities (Ming et al., 2013). It is an economically important crop widely used for food, medicinal, and ornamental purposes (Ming et al., 2013). As one of the top 10 most famous traditional flowers of China, sacred lotus has many prominent ornamental features, such as colorful and diverse corollas, a pleasant fragrance, and elegant plant architecture (Wang and Zhang, 2005). Plant architecture is an important trait that directly affects the applications of sacred lotus (Wang and Zhang, 2005). As aquatic potted plants suitable for family cultivation, small architecture (SA) lotuses have received increasing attention over the past years (Zhang, 2011). On the other hand, large architecture (LA) lotuses can better adapt to deep-water cultivation and have great significance in the beautification and purification of waterscapes and wetland restoration and construction, and are thus in great demand (Zhang, 2011). Hence, the development of new lotus varieties with extreme plant architecture is an important direction in lotus breeding. However, the breeding process is greatly hindered by a lack of background knowledge regarding the genetic mechanisms underlying lotus plant architecture traits.

Plant architecture, which is determined by a number of morphological characteristics, refers to the complex three-dimensional organization of the aerial parts of plants (Wang and Li, 2006). Plant height, a decisive factor in plant architecture, is mainly constrained by stem elongation (Wang and Li, 2008). The elongating growth of plants is mainly controlled through the integration of cell division and cell expansion. These two processes affect cell number and cell size, respectively (Reinhardt and Kuhlemeier, 2002). Generally, variation in final organ size is highly positively correlated with cell number, whereas the relationship between cell size and organ size is not as strong (Dhondt et al., 2013). An increase in cell number may occur by either higher cell division rates or an extended cell proliferation phase (Dhondt et al., 2013). Similarly, alterations in cell size depend on cell expansion rates and the duration of cell expansion. Increased cell size requires structural modifications and extension of the cell wall (Gonzalez et al., 2009). The primary cell wall contributes to cell expansion during cell development, while the secondary cell wall enhances mechanical strength and fixes cell size (Boerjan et al., 2003). Both primary and secondary cell walls directly determine the final cell size (Boerjan et al., 2003). The plant architecture of the lotus is assessed by plant height, which is measured as the length of the petiole and peduncle according to the instructions of the International Nelumbo Registration Form and Guideline. In our previous work, an F1 segregation population was obtained from parents that differed remarkably in plant architecture. The petiole length and peduncle length of the F1 offspring exhibited normal distributions, indicating that these two characteristics are quantitative traits and controlled by many minor genes. It has been reported that petiole elongation in response to submergence and phytohormones was mainly caused by increased cell number, whereas cell length showed few changes or even decreased, indicating that the elongation of lotus petioles under submergence is mainly caused by cell division (Jin et al., 2017). However, little is known about the morphological and genetic mechanisms underlying lotus plant architecture.

A key prerequisite for the genetic characterization of quantitative traits is the availability of a large quantity of genetic markers across the genome. Next generation sequencing has provided a rapid and effective strategy for the identification of genetic variations. With the release of increasing amounts of genomic sequencing data, whole genome re-sequencing (WGRS) has been successfully applied in the identification of genome-wide variants in many plant species (Xu and Bai, 2015). In the chickpea, based on WGRS data from 16 ascochyta blight (AB)-resistant and 24 AB-susceptible genotypes, fixation index (F_ST_) genome scan identified a 100-kb region that showed the largest F_ST_ on chromosome 4. This region exactly overlapped the genomic region previously detected by genome-wide association studies (GWAS) and quantitative trait loci (QTL) mapping (Li et al., 2017). In the rice, WGRS was performed on 11 preharvest sprouting (PHS) susceptible accessions and 10 PHS resistant accessions, and approximately 20,000 PHS-associated single nucleotide polymorphisms (SNPs) showing distinct allelic distributions between the two groups were detected. Some regions containing high numbers of these SNPs overlapped with previously reported QTLs associated with PHS (Lee et al., 2017). In the peach, chi-square test identified two SNPs in a fruit size candidate gene showing distinct allelic distributions between 30 small and 30 large fruit peach varieties (Cao et al., 2016). These significantly differentiated variants between groups with contrasting phenotypes can serve as molecular markers in future breeding programs (Cao et al., 2016).

The genome sequences of two lotus individuals have been released (Ming et al., 2013; Wang et al., 2013), enabling WGRS in the lotus. At present, WGRS has only been applied in evolutionary studies on 19 lotus germplasms and in detecting genome-wide simple sequence repeat (SSR) markers by WGRS in ‘Chiang Mai wild lotus’ (Hu et al., 2015; Huang et al., 2018). However, few trait-associated variants have been identified in the lotus. In the present study, 43 representative lotus germplasms with stably contrasting plant architecture were selected based on phenotypic measurement of 390 lotus germplasms for three consecutive vintages (2013–2015). Differences in cell number and size between LA and SA lotuses were detected using histological analysis. Highly differentiated genomic regions and variants between LA and SA lotus groups were then identified through F_ST_ genome scanning and chi-square test following WGRS of 20 representative lotus germplasms. Expression analyses of candidates in highly differentiated genomic regions and validation of developed plant architecture-associated InDel markers in lotus supported the reliability of our results. Our discovery of highly differentiated genomic regions and genetic variants which might be related to plant architecture in the lotus will provide vital clues for unraveling the genetic basis of plant architecture, and promising functional markers in the lotus.

## Materials and Methods

### Plant Materials

The 390 lotus germplasms with diverse plant architectures were planted in separate cement pools in a trial plot of the China Lotus Research Center, Wuhan, Hubei, China. For each lotus germplasm, three rhizomes were planted in responding cement pool. Petiole length (cm) was measured from the mud surface to the joint of the petiole and leaf, and peduncle length (cm) was measured from the mud surface to the joint of the peduncle and flower. Five leaves and five flowers of each lotus germplasm were chosen randomly in each pool. Normality tests were performed on the phenotypic data of the two traits in each year using SPSS 19.0 software (SPSS, Inc., Chicago, IL, United States). Fresh young leaves from 43 lotus germplasms (**Supplementary Table 1**) that showed extreme plant architecture were collected, flash frozen in liquid nitrogen, and stored at -70°C. Genomic DNA was extracted using the cetyltrimethyl ammonium bromide (CTAB) method (Doyle, 1987) and evaluated by 1.2% (w/v) agarose gel electrophoresis and with a NanoDrop 2000 spectrophotometer (Thermo Fisher Scientific, Waltham, MA, United States).

### Paraffin Sectioning and Light Microscopy Analyses

For cell morphological measurements, the middle regions of the petioles and peduncles were harvested at the maturation stage from 10 LA and 10 SA lotus germplasms. Three petioles and peduncles were collected from each germplasm and fixed in 70% formaldehyde-acetic acid-ethanol for 24 h. Samples were then dehydrated in an ethanol series (Johansen, 1940) and embedded in paraffin wax. Ten 5-μm transverse and vertical sections per sample were cut with a Leica RM2235 rotary microtome, mounted, and stained with 1% safranin O and 0.5% Fast Green. Cells were measured under a NLCD500 light microscope using ScopeImage 9.0 software (Nanjing Jiangnan Novel Optics Co. Ltd., Nanjing, China). Epidermal cells were chosen to represent cells in the petioles and peduncles (Ridge and Amarasinghe, 1984; Allard and Nelson, 1991). The widths and lengths of 15 randomly selected epidermal cells were measured in transverse and vertical sections, respectively. The cell numbers of petioles/peduncles in the vertical axis were determined by the total length and average cell length of the petioles/peduncles. The data are presented as the means ± standard error of the mean of three biological replicates. For statistical analysis, Duncan’s multiple range test (*P* < 0.05) was selected for comparisons among lotus germplasms. The data were further subjected to linear regression modeling using SPSS 19.0 software to evaluate the relationships between cell morphological traits and plant height.

### WGRS and Read Alignment

Whole genome re-sequencing was then conducted separately on the 20 lotus germplasms used in the cell morphological analyses. The 20 libraries with a target insert size of 350 bp were prepared according to the Illumina protocol and sequenced using the Illumina HiSeq 4000 to generate 150-bp pair-end sequence reads. The raw sequence data are available in the National Center for Biotechnology Information sequence read archive with the data accession number SRP145546.

Before aligning reads to the lotus reference genome, quality control of the raw data was performed following strict filtering criteria for removing paired reads when: (a) the percentage of unidentified nucleotides (N) was more than 10% in either of the paired reads; (b) there were more than 10-nucleotide adapters in either of the paired-end reads; (c) there were more than 50% low-quality bases (Phred score ≤ 5) in either of the paired reads; and (d) putative PCR-duplicated reads were generated by PCR amplification during the library construction process.

The filtered high-quality reads from each sample were aligned to the *N*. *nucifera* reference genome^1^ (Ming et al., 2013) using Burrows-Wheeler Aligner (BWA) software (Li and Durbin, 2009) with the command “mem -t 4 -k 32 –M.” Then, SAMtools software (Li et al., 2009) was used to convert and index the mapping results to BAM files with the settings “–bS –t.” When multiple read pairs had identical external coordinates, only the pair with the highest mapping quality was retained to improve the alignment result.

### Detection, Annotation, and Experimental Validation of SNPs and InDels

SNP and InDel calling was performed on the 20 re-sequenced germplasms using SAMtools (Li et al., 2009). The “mpileup” command was used to identify SNPs and InDels with the parameters “-m 2 -F 0.002 -d 1000” (Li et al., 2009). Raw SNP and InDel data were then filtered following strict criteria: (a) mapping quality ≥20; (b) coverage depth of the variant position ≥4 and ≤1000; (c) biallelic SNPs or InDels; and (d) rate of missing data within each group <50%. SNPs and InDels were further annotated using the ANNOVAR package (Wang et al., 2010).

To validate the reliability of variant calling, 35 SNPs (from 16 loci) and 35 InDels (at least 5 bp in length) were randomly selected. Primers (**Supplementary Table 2**) were designed according to the flanking sequences of the loci using Primer3^2^. PCR amplifications were carried out in a total volume of 15 μL containing 1 μL gDNA (50 ng/μL), 1.2 μL each of forward and reverse primers (10 μM), 7 μL 2× Es Taq Master Mix for PAGE (CWBIO, Beijing, China), and 4.6 μL ddH_2_O. PCR amplifications were performed on a 9902 Thermal Cycler (Applied Biosystems, Inc., Foster City, CA, United States) with the following reaction conditions: (1) denaturation at 94°C for 5 min; (2) 30 cycles of denaturation at 94°C for 30 s, annealing at the corresponding melting temperature of each primer (**Supplementary Table 2**) for 30 s, and extension at 72°C for 30 s; and (3) final extension at 72°C for 5 min. The PCR products of the SNPs were separated by agarose gel electrophoresis, purified, recovered using PCR purification kits (TSINGKE, Beijing, China), and sequenced on an ABI 3730xl sequencer (Applied Biosystems, Inc., Foster City, CA, United States). At each SNP locus, the sequences of all 20 germplasms obtained by Sanger sequencing were aligned using BioEdit version 7.0.5 software^3^. The PCR products of the InDels were separated using 6% polyacrylamide gel electrophoresis (PAGE) and visualized by silver staining. Allele size was estimated using a 20-bp DNA ladder (Dongsheng Biotech, Guangzhou, China). The amplified fragments for each germplasm and primer pair were scored manually as present (1) or absent (0). Using the sequence alignment and electrophoresis results, the genotypes of each SNP and InDel were retrieved for the 20 re-sequenced lotus germplasms.

Using the genome-wide 7,784,623 SNPs, genetic distances of the 20 lotus germplasms was calculated applying the p-distance method. The neighbor-joining (NJ) phylogenetic tree was constructed with the software TreeBestv1.9.2^4^ based on 1,000 bootstrap steps.

### Detection of Differentiated Genomic Regions/Alleles Related to Plant Architecture of Lotus

F_ST_ genome scan was used to screen the lotus genome for differentiated genomic regions between the LA and SA groups with the sliding window method (Axelsson et al., 2013). F_ST_ values between the LA and SA lotus groups were calculated with a window size of 50 Kb and a step size of 5 Kb using the VCFtools program (Danecek et al., 2011; Axelsson et al., 2013) and then Z-transformed (Axelsson et al., 2013). Highly differentiated genomic regions between the LA and SA lotus groups were extracted from windows with Z-transformed F_ST_ scores (ZF_ST_) ≥ 3, considering the low genomic differentiation between LA and SA lotus groups and to avoid false positive results (Kim et al., 2016). The highly differentiated genomic regions accounted for about 1.37% of the lotus genome and covering approximately 0.84% of genes on the lotus genome. The predicted functions of the genes located in these regions were obtained based on the gene annotation results of the lotus reference genome (Ming et al., 2013).

Gene Ontology (GO) enrichment analysis was processed by GOSeq and P values were calculated in Wallenius method (Young et al., 2010). Enriched GO terms were classified into categories by cellular component, molecular function, biological process. To minimize the rate of false positives, *P*-values were corrected with Benjamini–Hochberg FDR (false discovery rate) correction (Benjamini and Hochberg, 1995). Only terms with a corrected *P*-value < 0.05 were considered significant.

The genome-wide genotype frequencies of the reference genotype and alteration genotype for each SNP and InDel were separately counted in the LA and SA groups, and then chi-square test was used to analyze the differences in genotype frequency distribution between the LA and SA groups for each SNP and InDel (Zhang et al., 2016). SNPs and InDels were considered significantly differentiated between the LA and SA lotus groups when the corrected *P* < 0.01. Among these variants, the SNPs and InDels located in the highly differentiated genomic regions were considered putative variants responsible for the regulation of lotus plant architecture. Genes showing at least one change at the amino acid level were considered candidates.

### Expression Profiles of Candidate Genes in Two Lotus Germplasms With Contrasting Plant Architecture

The expression patterns of candidate genes were explored using quantitative reverse-transcription PCR (qRT-PCR) at four development stages during petiole growth in LA lotus ‘Chongtai’ and SA lotus ‘Honghehuan.’ Total RNA was extracted from petiole tissues using an OminiPlant RNA Kit (CWBIO, Beijing, China). The quality and quantity of the RNA was examined using 1.2% (w/v) agarose gel electrophoresis and a NanoDrop 2000 spectrophotometer (Thermo Fisher Scientific, Wilmington, DE, United States), respectively. Reverse transcription to cDNA was carried out with 1 μg total RNA using the All-in-One First-Strand cDNA Synthesis Kit (Yugong Biolabs, Lian Yungang, China). The specific primers of the candidate genes were designed using Primer3 software (see footnote 2) (**Supplementary Table 3**). Actin was used as the internal control with forward and reverse primers GCGTTCTGCCGTCTTCTAAA and CCCTCTTGGATTGTGCCTC, respectively.

qRT-PCR was performed in 384-well plates, with a total volume of 20 μL per well: 10 μL Hieff qPCR SYBR Green Master Mix (Low Rox Plus) (YESEN, Shanghai, China), 0.4 μL of each of forward and reverse primers, 1 μL cDNA template, and 8.2 μL ddH_2_O. The qRT-PCR reactions were performed in triplicate for all genes and internal controls. The reactions were performed on a QuantStudio 7 Flex Real-Time PCR System (Applied Biosystems, Inc., Foster City, CA, United States). Gene expression was presented as relative expression levels calculated using the 2^-ΔΔCT^ method (Livak and Schmittgen, 2001).

### Development of Lotus Plant Architecture-Related InDel Markers

InDels (at least 5 bp in length) located in the highly differentiated genomic regions with distinct allelic distribution between lotus LA and SA groups were selected to develop molecular markers. The verification methods were as described in part 4 of this section. Among the 43 representative germplasms, 11 LA and 12 SA lotus germplasms that were not re-sequenced were used in marker screening. Primers for the InDel markers are listed in **Supplementary Table 4**. The chi-square test was used to estimate the allelic differentiation of the developed InDel markers between the lotus LA and SA groups. For clustering analysis, similarity coefficients were used to construct an unweighted pair group mean algorithm (UPGMA) dendrogram using the SAHN module in NTSYS-pc v.2.10 software (Rohlf, 1998).

## Results

### Plant Architecture Phenotypic Characterization of 390 Lotus Germplasms

**FIGURE 1:**
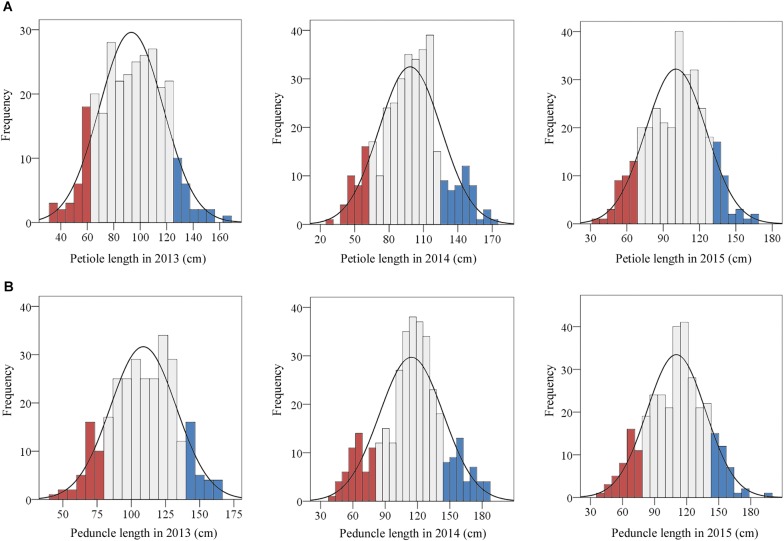
Frequency distributions of petiole and peduncle length in 390 lotus germplasms. The traits were evaluated in 2013, 2014, and 2015. **(A)** The frequency distributions of petiole length in 2013, 2014, and 2015. **(B)** The frequency distributions of peduncle length in 2013, 2014, and 2015. The blue and red bars represent the top and bottom 15%, respectively, of the frequency distributions of the traits.

In total, 390 representative lotus germplasms with diverse plant architecture were collected across China. The 390 lotus germplasms are modern cultivars with diverse plant architecture phenotypes. Because the plant architecture of the lotus was mainly determined by petiole and peduncle length, the two traits were further measured in these lotus germplasms for three consecutive vintages (2013–2015). The phenotypic data of the two traits followed a normal distribution across the 3 years based on the results of the normality test (**Figure 1**). As shown in **Figure 1**, 21 LA lotus germplasms (blue bars) were located in the top 15% of the phenotypic distributions, while 22 SA lotus germplasms (red bars) were located at the bottom 15% of the phenotypic distributions in all 3 years. Details on the 43 lotus germplasms are listed in **Supplementary Table 1**. To further explore the morphological characteristics and genomic differences between LA and SA lotuses, the 21 LA lotus germplasms together with the 22 SA lotus germplasms were chosen for further analyses, as the two groups expressed divergent plant architecture phenotypes.

### Cell Morphology Characteristics of LA and SA Lotuses

**FIGURE 2:**
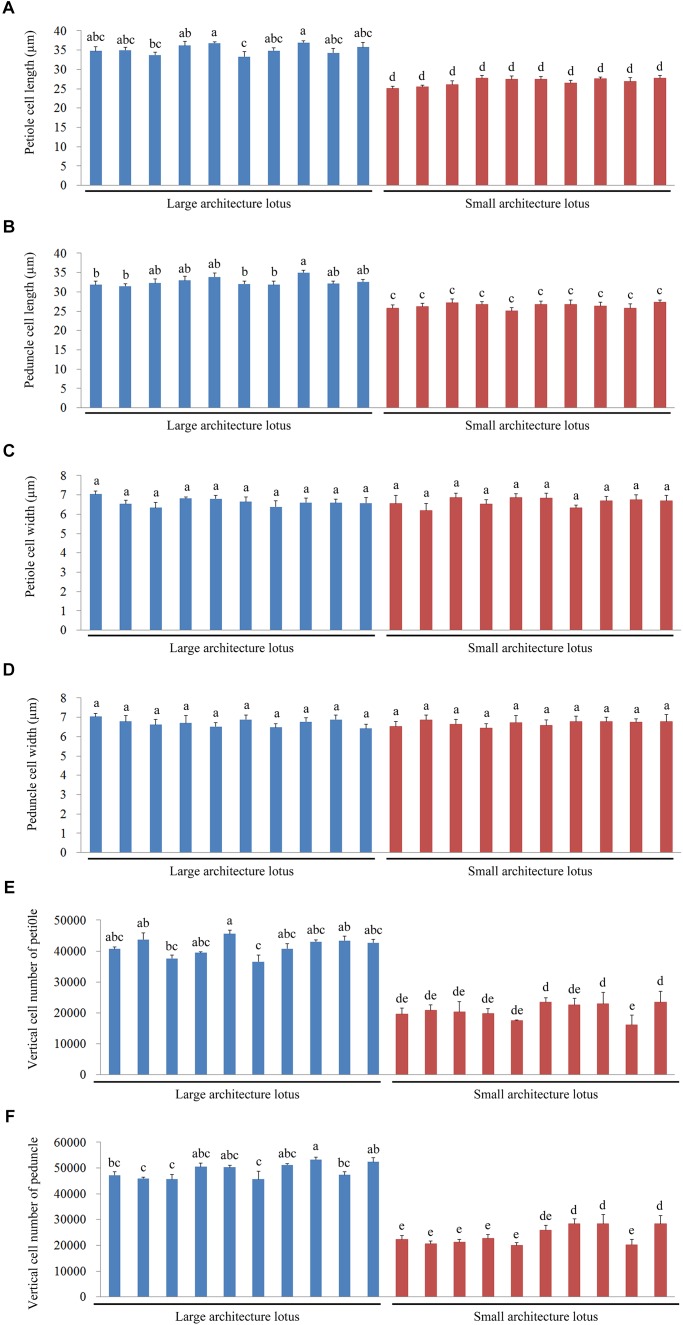
Comparisons of cell length, cell width, and vertical cell number of the petiole and peduncle among 10 large architecture (LA) and 10 small architecture (SA) lotus germplasms. Values are the means ± standard error (SE) with three replicates per column. **(A)** The lengths of petiole cells. **(B)** The lengths of peduncle cells. **(C)** The widths of petiole cells. **(D)** The widths of peduncle cells. **(E)** The numbers of petiole cells in the vertical direction. **(F)** The numbers of peduncle cells in the vertical direction. Blue columns represent the 10 LA lotus germplasms, and red columns represent the 10 SA lotus germplasms.

As mentioned above, cell number and cell size are two vital factors that facilitate elongation growth in plants (Reinhardt and Kuhlemeier, 2002). A total of 10 LA and 10 SA germplasms were randomly selected from the 43 representative lotus germplasms. All the 20 lotus samples are temperate lotus and are supposed to have no kinship according to the existing authoritative record (Wang and Zhang, 2005; Zhang, 2011). The variations in cell length, cell width, and verticle cell number of the petioles and peduncles were then measured through paraffin sectioning and light microscopy (**Supplementary Figure 1**). Multiple comparative analyses of petiole cell lengths and widths indicated that the petiole cell length of LA lotuses was significantly longer than that of SA lotuses (**Figure 2A**), whereas there was no significant difference in cell width between the LA and SA lotuses (**Figure 2C**). Additionally, the number of petiole cells in the vertical direction was significantly higher in LA lotuses than in SA lotuses (**Figure 2E**). The phenotypic characteristics of peduncle cells also showed similar patterns to those of petiole cells, as suggested in **Figures 2B,D,F**.

**FIGURE 3:**
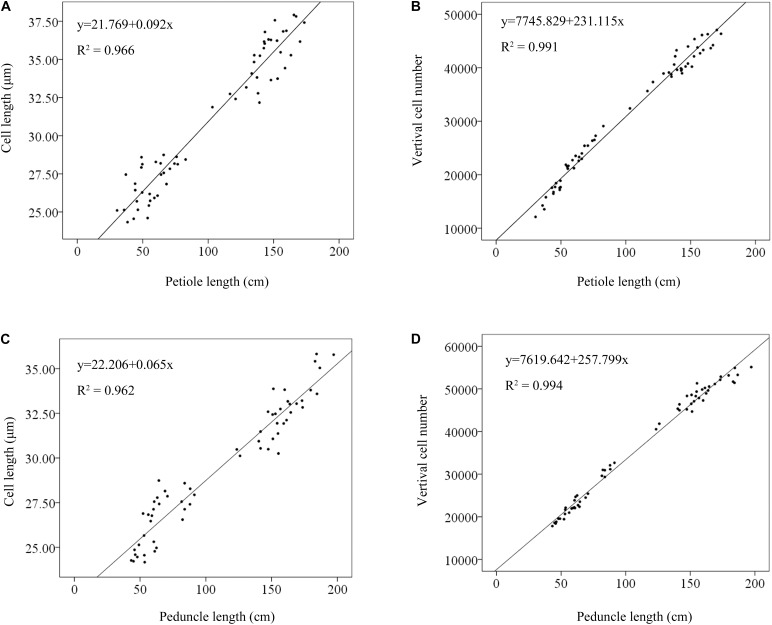
Linear regression analyses of the relationships between cell length and petiole length **(A)**, vertical cell number and petiole length **(B)**, cell length and peduncle length **(C)**, and vertical cell number and peduncle length **(D)**. The solid lines were fitted by regression. Phenotypic data from 10 LA and 10 SA lotus germplasms were used in the linear regression analysis.

Furthermore, to determine the main causes that may be responsible for variation in plant height, the relationships between verticle cell number and petiole/peduncle length and between cell length and petiole/peduncle length were evaluated using linear regression analysis. The linear relationship between cell number and petiole length (*R*^2^ = 0.991; **Figure 3B**) was stronger than that between cell length and petiole length (*R*^2^ = 0.966; **Figure 3A**). The linear relationship between cell number and peduncle length was strong (*R*^2^ = 0.994; **Figure 3D**) and slightly larger than that between cell length and peduncle length (*R*^2^ = 0.962; **Figure 3C**). The results indicated that petiole and peduncle length had strong linear relationships with cell number and cell length.

### WGRS and Genome-Wide Variant Detection in the 20 Lotus Germplasms With Contrasting Plant Architecture

To detect genome-wide genetic variations between LA and SA lotuses, WGRS was conducted in the 10 LA and 10 SA lotus germplasms (**Supplementary Table 1**) used in the cell morphological analyses. Whole genome sequencing of the 20 selected lotus germplasms yielded 197.29 Gb of raw data (**Table 1**). A total of 196.12 Gb of high-quality clean data was obtained from the 20 germplasms following appropriate quality filtering as described in the “Materials and Methods” Section. The amount of high-quality clean data varied from 8.70 to 11.59 Gb for each germplasm (**Table 1**). The average sequencing depth was 12.22×. On average, 97.76% of the high-quality reads were successfully mapped onto the lotus reference genome using BWA software (Li and Durbin, 2009) (**Table 1**). Mapped reads of the 20 germplasms covered approximately 95.66–98.62% of the reference sequence (**Table 1**), indicating that the generated data set was highly relevant to the reference genome.

**Table 1:** Summary of the re-sequencing data from 20 lotus germplasms.

Sample name	Raw data (bp)	High-quality data (bp)	Total reads mapped (%)	Sequencing depth (X)	≥1× coverage
Chongtai lotus	9,332,930,700	9,299,393,400	97.73	11.88	97.74
Qianban lotus	10,335,317,700	10,302,705,900	98.07	12.86	98.49
Xingkongmudan	9,938,182,800	9,898,820,100	97.89	12.34	97.16
Fengjuanhongqi	8,730,950,400	8,700,571,500	97.82	11.07	96.61
Yuchan	9,218,801,100	9,183,069,300	98.16	11.58	97.21
Xiangbihe	9,150,490,500	9,114,393,300	97.40	11.56	97.28
Shuguangxiu	9,420,146,700	9,388,740,600	97.90	11.83	96.49
Donghuxinhong	9,874,330,800	9,846,971,100	98.09	12.45	97.08
Hongtai lotus	9,155,348,400	9,130,642,200	98.18	11.44	98.12
Number 689	10,176,733,800	10,123,365,300	97.77	12.82	95.66
Jinlingzhixing	10,802,341,200	10,769,308,500	97.95	13.32	97.90
Danyue	9,808,241,100	9,774,771,900	97.31	11.90	97.36
Zuidongfeng	10,461,610,800	10,426,700,100	98.16	12.87	98.62
Hongyun	11,131,869,900	11,098,322,100	98.21	13.56	98.25
Xiaoqu	10,054,341,600	10,009,623,900	98.03	12.50	97.34
Xiaojingling	9,525,178,800	9,502,553,100	96.97	11.60	97.61
Hong Hehuan	9,148,683,300	9,126,764,700	96.90	11.27	96.79
Jinlinghuodu	11,620,749,300	11,586,919,500	97.80	14.13	97.81
Qinhuaibaiyu	9,338,316,900	8,798,182,800	97.21	11.14	97.23
Hongchong	10,066,581,900	10,037,385,000	97.65	12.37	97.82
Total	197,291,147,700	196,119,204,300	–	–	–
Average	9,864,557,385	9,805,960,215	97.76	12.22	97.43

Genome-wide SNPs and InDels were detected among the 20 re-sequenced germplasms by applying SAMtools software (Li et al., 2009). To increase the accuracy and minimize the false-positive rates of detected SNPs and InDels, four filtering criteria were applied as described in the “Materials and Methods” section. A total of 7,784,623 SNPs and 1,103,064 InDels were detected (**Supplementary Table 5**) with an estimated SNP and InDel density of 9.68 and 1.37 per Kb. Among these polymorphic SNPs and InDels, 4,948,436 (63.57%) SNPs and 654,238 (59.31%) InDels were detected in intergenic regions, 477,162 (6.13%) SNPs and 110,722 (10.04%) InDels were detected in upstream and downstream regions, and 2,355,772 (30.26%) SNPs and 336,134 (30.47%) InDels were detected in genic regions. Among the polymorphisms in genic regions, 264,445 SNPs and 7,994 InDels were in exonic regions, and 2,090,709 SNPs and 327,807 InDels were in intronic regions (**Supplementary Table 5**).

To validate the quality of the identified SNPs, 35 SNPs (from 16 loci) and 35 InDels were randomly selected for experimental validation. The locations and primers of SNPs and InDels selected in each scaffold are presented in **Supplementary Table 2**. Of the 35 SNPs, 31 were successfully sequenced and 98.23% contained the predicted genotypes (**Supplementary Table 2** and **Supplementary Figure 2**). Of the 35 InDels, 30 were confidently scored and 96.17% contained the predicted genotypes (**Supplementary Table 2** and **Supplementary Figure 3**). These results revealed fairly high validation rates and confirmed the high quality of the SNPs and InDels detected in this study.

The phylogeny tree of the 20 lotus germplasms was further conducted based on all the SNP sites (**Supplementary Figure 4**). A genetic differentiation can be observed between LA group and SA group of lotuses. Some LA lotuses were genetically closer with SA lotus group. The reason maybe that the 20 lotus germplasms are all modern germplasms, and there may be genetic introgression between the two lotus groups.

### Detection of Candidate Genomic Regions and Variations in Lotus Plant Architecture

**FIGURE 4:**
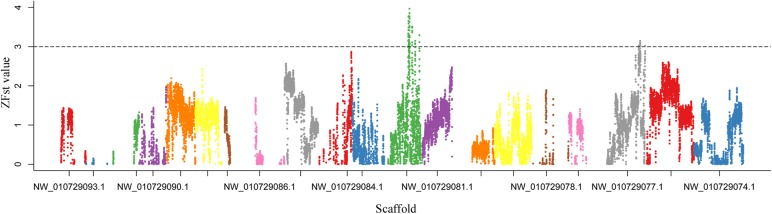
Distribution of Z-transformed fixation index (ZF_ST_) values between LA and SA lotus groups. ZF_ST_ values were plotted along lotus scaffolds, which are presented in different colors. ZF_ST_ values above the dashed horizontal line (ZF_ST_ ≥ 3) indicate genomic regions with extreme genetic differentiation between LA and SA lotus groups.

F_ST_ genome scan is an effective method which uses of large numbers of molecular markers to scan regions with extreme genetic differentiation between divergent groups (Holsinger and Weir, 2009; Fumagalli et al., 2013). To detect highly differentiated genomic regions between the LA and SA lotus groups, F_ST_ values were calculated between the two groups in 50-Kb windows with 5-Kb steps across the genome. Overall, F_ST_ values ranged from -0.058 to 0.591 with an average value of 0.074, which indicated the close relationship between the two groups (**Supplementary Figure 5**). **Figure 4** shows the ZF_ST_ distribution of the sliding windows across the lotus genome. Considering the low genomic diversity between the LA and SA lotus genotypes, a strict cutoff parameter ZF_ST_ ≥ 3 was applied to select highly differentiated genomic regions which putatively related to lotus plant architecture. In total, 1,388 windows were identified, distributed across 35 scaffolds (**Supplementary Table 6**). After integration of these windows, a total of 92 regions covering 11.02 Mb of the genome were obtained. The highly differentiated genomic regions accounted for 1.37% of the lotus genome and the largest region was located on scaffold NW_010729262.1, covering 635 Kb. A total of 238 genes were identified within these highly differentiated genomic regions (**Supplementary Table 6**).

To better understand the function of the genes in differentiated genomic regions, gene functional enrichment analysis was performed. In detail, 114 significantly overrepresented GO terms (corrected *P* < 0.05) were detected. Interestingly, several cell development and cell wall construction related GO terms were enriched. For example, GO: 0008360 was related to regulation of cell shape, and GO: 0022604 was regulation of cell morphogenesis). GO: 0010393 referred to galacturonan metabolic process, with GO: 0022603 is regulation of anatomical structure morphogenesis. These Go terms were related to cell proliferation/elongation (Kutschera and Niklas, 2013; Kalve et al., 2014; Jin et al., 2017). Meanwhile, GO: 0006511 and GO: 0016985 which are annotated as ubiquitin-dependent protein catabolic process and mannan endo-1,4-beta-mannosidase activity were also important for cell development and cell wall construction (Day et al., 2013; Kalve et al., 2014) (**Supplementary Table 7**).

Additionally, chi-square test was used to detect the allelic differentiation of SNPs and InDels between the LA and SA lotus groups. In total, 34,055 SNPs and 3,828 InDels showed distinct allelic distribution (corrected *P* < 0.01) between the LA and SA lotus groups (**Supplementary Figures 6, 7**). Among them, 17,154 SNPs and 1,554 InDels were located in the highly differentiated genomic regions (**Table 2**). Subsequently, we placed more emphasis on variants that had effects on amino acids, as these variants are more important in functional analyses. A total of 120 SNPs together with six InDels were predicted to cause amino acid changes in 60 genes (**Supplementary Table 8**).

**Table 2:** Summary of the single nucleotide polymorphisms (SNPs) and insertions and deletions (InDels) that showed distinct allelic distribution in the differentiated genomic regions between large architecture (LA) and small architecture (SA) lotus groups.

Category	Number of SNPs	Category	Number of InDels
Upstream	306	Upstream	56
Exonic_Stop gain	3	Exonic_Stop gain	1
Exonic_Synonymous	88	Exonic_Frameshift deletion	2
Exonic_Non-synonymous	117	Exonic_Frameshift insertion	2
Intronic	2,332	Exonic_Non-frameshift insertion	1
Splicing	1	Intronic	342
Downstream	266	Downstream	48
Intergenic	14,041	Intergenic	1,102
Total	17,154	Total	1,554

### Expression Analysis of the Candidate Genes Putatively Related to Plant Architecture in Lotus

Among the 60 genes with amino acid changes, 19 could not be annotated using the known annotation databases. A complete list of the remaining 41 genes is provided in **Supplementary Table 8**. Some genes were predicted to be associated with cell proliferation. *LOC104587827*, predicted to be a small glutamine-rich tetratricopeptide repeat-containing protein, and *LOC104598313*, annotated as receptor-like protein 12, have been suggested to be involved in the cell cycle (Rodrigues et al., 2013; Xue et al., 2013). *LOC104598259*, a probable zinc finger protein ZPR1 homolog, the deficiency of which blocks phase progression and arrests the cell cycle, has been reported to be involved in cell proliferation (Gangwani, 2006). *LOC104609670* (putative G-type lectin S-receptor-like serine/threonine-protein kinase At1g61610) may be involved in the cell cycle as the target gene of BABY BOOM AP2/ERF domain protein, which causes cells to re-enter the cell cycle (Passarinho et al., 2008). *LOC104589899* is putative rRNA-processing protein EBP2, and EBP2 has been suggested to be involved in cell proliferation (Jeon et al., 2015). Virus-induced gene silencing of EBP2 in *Nicotiana benthamiana* resulted in small plant architecture (Jeon et al., 2015).

Several cell wall construction-related genes were also detected. *LOC104593723* and *LOC104591005* are both polygalacturonase-like genes that function as cell wall hydrolytic enzymes and act as positive regulators of cell growth (Daher and Braybrook, 2015). *LOC104598294* (CMP-sialic acid transporter 4-like) may play a role in cell wall composition and structure and in the cross-talk between the auxin, SA, and abiotic stress signaling pathways (Cakir and Olcay, 2013). *LOC104598378* was predicted to encode cellulose synthase-like protein D1, which plays important roles in cell wall formation and is essential for plant architecture development through the regulation of both cell division and cell expansion (Luan et al., 2011).

*LOC104598311* is predicted to be an aspartyl protease family protein, which caused severe dwarfism in *Arabidopsis* when mutated and overexpressed (Paparelli et al., 2012). *LOC104587816* is a fatty acid amide hydrolase-like gene, and T-DNA knockout mutation resulted in severe dwarfism whereas overexpression enhanced seedling growth in *Arabidopsis* (Wang et al., 2006).

**FIGURE 5:**
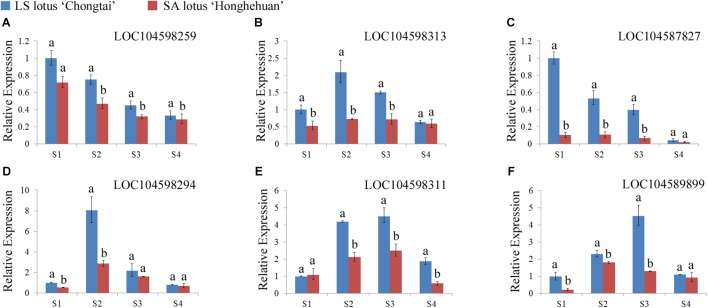
Expression profiles of the six candidate genes related to plant architecture regulation in the lotus. **(A–F)** represented the relative expression levels of the gene LOC104598259, LOC104598313, LOC104587827, LOC104598294, LOC104598311, and LOC104589899 at four representative developmental stages (S1–S4) in the petioles of LA lotus ‘Chongtai’ and SA lotus ‘Honghehuan’ via quantitative reverse-transcription PCR, respectively.

Six genes among these candidates were selected for qRT-PCR analyses to determine whether they have different expression modes in lotuses with opposite plant architecture. The expression profiles of the six candidate genes were analyzed in four petiole developmental stages in the LA lotus ‘Chongtai’ and SA lotus ‘Honghehuan.’ Four genes (*LOC104598259*, *LOC104598313*, *LOC104587827*, and *LOC104589899*), which are predicted to be involved in the cell cycle, exhibited much higher expression in ‘Chongtai’ than in ‘Honghehuan’ during the S1, S2, and S3 stages (**Figures 5A–C**, F). In addition, expression levels of the cell wall-related gene *LOC104598294* in the S1 and S2 stages were also significantly higher in ‘Chongtai’ than in ‘Honghehuan,’ whereas the expression difference was not significant in stage S3 (**Figure 5D**). Similarly, expression of the carbohydrate metabolism-related gene *LOC104598311* was significantly upregulated in the S2, S3, and S4 stages in ‘Chongtai’ (**Figure 5E**).

### Verification of Candidate InDel Markers Associated With Plant Architecture in the Lotus

**FIGURE 6:**
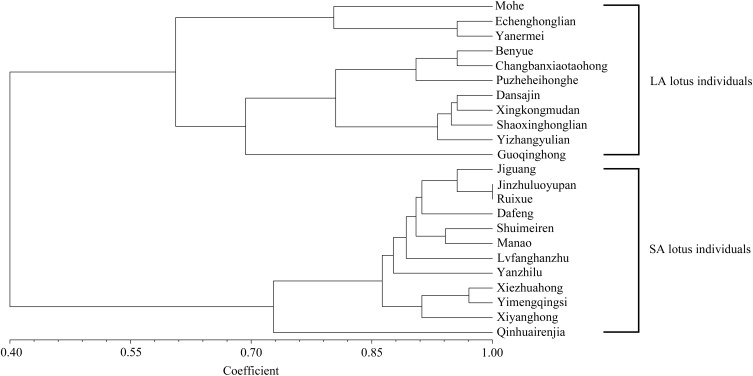
Unweighted pair group mean algorithm (UPGMA) phylogenetic tree of 11 LA and 12 SA lotus germplasms based on similarity coefficients using 34 new InDel markers associated with lotus plant architecture.

A total of 386 InDels (at least 5 bp in length) located in the highly differentiated genomic regions showing distinct allelic distribution between LA and SA lotus groups were screened out. To develop InDels markers related to lotus plant architecture, 70 InDels were randomly selected and verified in the 11 remaining LA and 12 remaining SA lotus germplasms that were not re-sequenced among the 43 representatives of extreme plant architecture. Following PAGE validation, three primer pairs that did not amplify any fragments, 16 primer pairs that amplified numerous non-target bands or smeared target bands, and one marker that was shown to be monomorphic were abandoned. The remaining 50 InDels markers were confidently scored and exhibited dimorphism (**Supplementary Table 4** and **Supplementary Figure 8**). Chi-square test further indicated that 34 of the 50 InDels differed significantly (*P* < 0.01) between the LA and SA lotus groups, indicating the stability of the significant association between these InDel markers and plant architecture type (**Supplementary Table 4**). Despite the close association with plant architecture, the 34 new InDel markers were also examined for their potential use in genetic diversity analyses among the 23 lotus germplasms. The UPGMA phylogenetic tree revealed two major clusters, with 11 LA lotus germplasms in one cluster and 12 SA lotus germplasms in the other (**Figure 6**), indicating that the 34 InDel markers can be applied to differentiate LA and SA lotuses.

## Discussion

### Critical Cellular Factors Affecting Plant Architecture Variations in Lotus

The plant architecture of the lotus is mainly determined by plant height, which is quantified as the length of the petiole and peduncle according to the International Nelumbo Registration Form and Guideline. Variations in organ size are usually ascribed to cell number and cell size, which are mainly determined by cell division and cell expansion processes (Reinhardt and Kuhlemeier, 2002).

We found that petiole length had a strong linear relationship with vertical cell number (*R*^2^ = 0.991), as did peduncle length (*R*^2^ = 0.994) (**Figure 3**). Additionally, the vertical cell numbers of the petiole and peduncle in LA lotuses were significantly higher than those in SA lotuses (**Figure 2**). The results were consistent with previous studies showing that petiole elongation in response to submergence and phytohormone treatment was mainly caused by increases in cell number (Jin et al., 2017). Because cell number is mainly determined by the process of cell division, we suggested that the promotion of cell division plays an important role in petiole elongation in the lotus. The cell length of the petiole and peduncle in LA lotuses was significantly longer than that in SA lotuses (**Figure 2**). Although the cell length of the petiole and peduncle showed strong linear relationships with petiole length (*R*^2^ = 0.966) and peduncle length (*R*^2^ = 0.962), respectively (**Figure 3**), the linear relationship between cell length and plant height was relatively weaker than that between vertical cell number and plant height. In a previous study, cell length showed few changes or even decreased when petioles were elongated in response to submergence and treatment with different phytohormones (Jin et al., 2017). These results indicate that cell elongation may also contribute to variations in lotus plant architecture, but its role in lotus plant height management was smaller than that of cell division. Thus, we suggest that both cell division and cell elongation contribute to variations in plant architecture in the lotus, and cell division seems to play a more important role than cell elongation.

### WGRS for Detecting Genome-Wide DNA Polymorphisms and Genomic Regions Highly Differentiated Between LA and SA Lotus Groups

Whole genome re-sequencing has been successfully applied to detect candidate genomic regions and alleles associated with complex traits from diverse genetic backgrounds (Lee et al., 2017; Li et al., 2017). Based on continuous phenotype measurement results of 390 lotus germplasms from 2013 to 2015, 10 LA and 10 SA lotus germplasms were selected from 43 representatives showing extreme plant architecture phenotypes, and WGRS was performed to detect genome-wide DNA polymorphisms among the 20 selected lotus germplasms. The average sequencing depth of the 20 high-quality datasets reached 12.22×, which was much deeper than the average sequencing depth of 4× reported in previous GWRS studies in the lotus (Huang et al., 2018). The deep sequencing depth ensured a high level of confidence in the detected DNA polymorphisms. A total of 31 SNPs and 30 InDels were validated using Sanger sequencing and PAGE analysis. The results indicated that 98.23% of the SNP genotypes and 96.17% of the InDel genotypes contained the predicted genotypes. The validation rate was higher than the 81.5% for SNPs obtained in “Chiang Mai wild lotus” and the 93% for InDels obtained in rice (Hu et al., 2015; Liu et al., 2015), which indicated the high quality of the identified SNPs and InDels. These abundant and reliable sequence variants provided a foundation for the detection of divergent genomic regions and alleles between LA and SA lotus groups that may be associated with plant architecture regulation.

The detection of genome-wide DNA polymorphisms has been reported as an effective tool for dissecting the genetic mechanisms of a specific trait (Jie et al., 2017). F_ST_ genome scan was conducted to identify highly differentiated genomic regions between the LA and SA lotus groups. After setting a strict cutoff parameter of ZF_ST_ ≥ 3, we obtained 92 highly differentiated regions covering an 11.02-Mb genome region accounting for 1.37% of the lotus reference genome. This is the first time that genomic regions putatively related to plant architecture of the lotus were identified based on WGRS data. Interestingly, several cell development and cell wall construction related GO terms were enriched based on the GO enrichment analyses of the 238 genes located in the plant architecture related genomic regions. Chi-square test detected 34,056 SNPs and 3,832 InDels that showed distinct allelic distributions between the LA and SA lotus groups. More than 80% of the highly differentiated SNPs and 75.18% of the InDels were located in non-genic regions, with lower distribution in exons, which is consistent with the SNP and InDel distribution patterns reported in rice (Lee et al., 2017). Meanwhile, there were more amino acid-altering SNPs (256 SNPs) than InDels (16 InDels) in the exonic regions, which can be explained by the fact that InDels are more deleterious than SNPs in exon regions, as InDels can cause frameshift mutations and amino acid substitutions that cause major changes in gene function (Clark et al., 2007; Lai et al., 2010).

Since only 20 sample were provided for the detection of plant architecture related regions, the loss of positive regions/alleles or the inclusion of negative regions/alleles related to plant architecture may be possible. An F1 segregation group of lotus plant architecture had been established in our group, the variants with distinct allelic distributions between the lotus LA and SA groups which located at the highly differentiated genomic regions are being applied for linkage analyses. Verification of the InDel markers in larger lotus germplasm samples is important to ensure the accuracy of these markers (Rubin et al., 2010; Axelsson et al., 2013). Meanwhile, we will conduct transformation system of lotus/Arabidopsis experiments in the subsequent experiment based on the candidate genes selected. Functional verification should be much more accurate and effective. It is our hope to combine the subsequent experimental data to achieve the maker assisted breeding of this important trait in lotus.

### Analysis of Candidates and Functional Polymorphisms Associated With Lotus Plant Architecture

The availability of the annotated reference genome allowed us to identify candidate genes and alleles underlying the highly differentiated genomic regions conferring lotus plant architecture. A total of 238 predicted genes were found in these genomic regions. Among them, 60 genes contained variants that caused amino acid changes and showed distinct allelic distribution between LA and SA lotus groups. Among 41 genes with functional annotations, five candidate protein-coding genes were predicted to be involved in cell proliferation. These genes were expected to be linked with plant architecture of the lotus, as cell division can influence the total number of cells in the petioles and peduncles and therefore the final organ size. Four genes were predicted to be related to cell wall construction. These genes were considered to be linked with plant architecture of the lotus, because the synthesis of primary and secondary cell wall formation can affect the cell elongation process and determine the final cell size (Boerjan et al., 2003). These genes are consistent with the results revealed in the aforementioned cell morphology study. One gene involved in carbohydrate metabolism and one gene involved in fatty acid amide metabolism were suggested to be involved in plant architecture regulation. In total, 11 candidates were considered the most plausible genes involved in plant architecture in the causal region. The relative expression patterns of six of the candidate genes showed significantly higher expression levels in LA lotuses than in SA lotuses at different developmental stages of the petiole, confirming the functions of these genes in the regulation of plant architecture in the lotus. Both the functional annotation and expression results confirmed the precision of the localization of plant architecture loci in the lotus, suggesting the accuracy and power of the F_ST_ genome scan and chi-square test. In addition to the genes mentioned above, there were also many genes located in the highly differentiated genomic regions containing variants with distinct allelic distribution between LA and SA lotus groups. The biological functions of these genes also warrant further investigation.

The polymorphisms of the 70 InDels located at the highly differentiated genomic regions putatively related to lotus plant architecture were further detected in 11 representative LA and 12 representative SA lotus germplasms, and 50 InDel markers amplified clear target bands and exhibited dimorphism. A total of 34 InDel markers (68%) were still highly differentiated (*P* < 0.01) between the LA and SA lotus groups, indicating the tight relationship between these InDel markers and plant architecture. Furthermore, the UPGMA phylogenetic tree constructed with the 34 InDel markers completely distinguished the LA and SA lotus groups. These InDels are predicted to be promising markers linked to plant architecture and may be applied in the early selection of plant architecture in the lotus.

In summary, we detected 7,784,623 SNPs and 1,103,064 indels among 20 lotus germplasms with contrasting plant architecture. F_ST_ genome scan identified 11.02 Mb of highly differentiated genomic regions between the two groups. Meanwhile, chi-square test revealed 34,055 SNPs and 3,828 InDels with distinct allelic distribution between the LA and SA lotus groups. Functional analyses revealed 11 candidate genes putatively associated with plant architecture management. In addition, we developed and verified 34 InDels markers putatively associated with plant architecture in the lotus. The candidate genes and trait-associated markers detected in this study will contribute to selective breeding and a greater understanding of the genetic mechanisms underlying plant architecture in the lotus.

## Author Contributions

L-QC and JZ conceived and designed the experiments. MZ and J-XY performed the experiments. JZ and MZ analyzed the data and wrote the paper. MZ, T-YM, H-HZ, and LX contributed reagents, materials, and analysis tools.

## Conflict of Interest Statement

The authors declare that the research was conducted in the absence of any commercial or financial relationships that could be construed as a potential conflict of interest.

## Footnotes

1 ftp://ftp.ncbi.nlm.nih.gov/genomes/refseq/plant/Nelumbo_nucifera/all_assembly_versions/GCF_000365185.1_Chinese_Lotus_1.1

2 http://bioinfo.ut.ee/primer3-0.4.0/primer3/

3 http://www.mbio.ncsu.edu/bioedit/page2.html

4 http://treesoft.sourceforge.net/treebest.shtml
