# Supplementary material for: Detection of Highly Differentiated Genomic Regions Between Lotus (Nelumbo nucifera Gaertn.) With Contrasting Plant Architecture and Their Functional Relevance to Plant Architecture
Source: Front Plant Sci. 2018 Aug 20;9:1219. doi: 10.3389/fpls.2018.01219 (PMC6110191; doi:10.3389/fpls.2018.01219)
Supplement: Supplementary file 1 [file Data_Sheet_1.DOCX]

***Supplementary Material***

**Detection of highly differentiated genomic regions between lotus (*Nelumbo nucifera* Gaertn.) with contrasting plant architecture and their functional relevance to plant architecture**

**Mei Zhao^1^, Ju-Xiang Yang^1^, Tian-Yu Mao^1^, Huan-huan Zhu^1^, Lin Xiang^1^, Jie Zhang^1*^, Long-Qing Chen^2*^**

^1^Key Laboratory of Horticultural Plant Biology, College of Horticulture and Forestry Sciences, Huazhong Agricultural University, Ministry of Education, Wuhan, China

^2^Southwest Engineering Technology and Research Center of Landscape Architecture (State Forestry Administration), Southwest Forestry University, Kunming, China

* **Correspondence:**

Jie Zhang

flybebrave@mail.hzau.edu.cn

Long-Qing Chen

chenlq@mail.hzau.edu.cn

**Supplementary Figures**

**
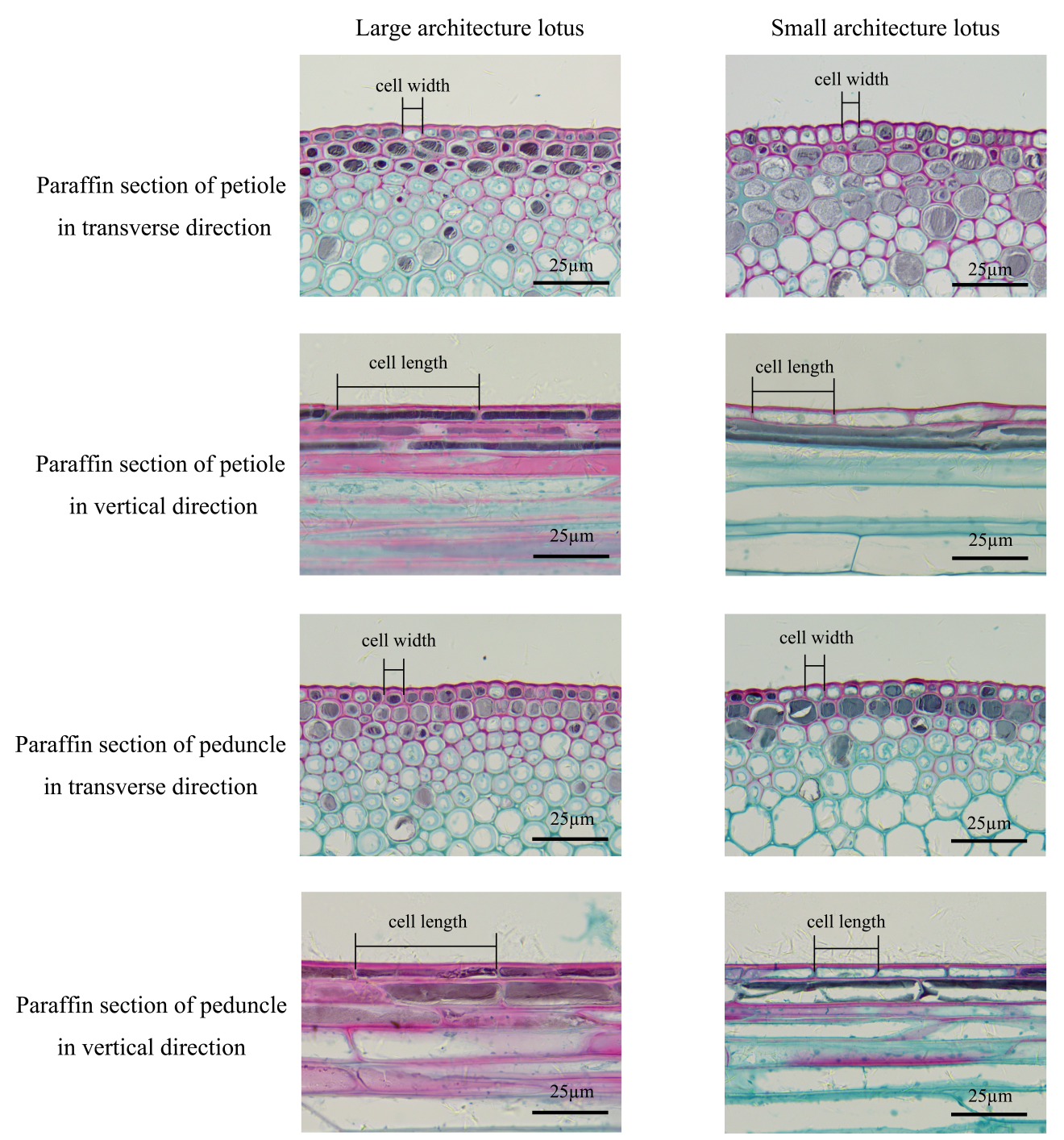
**

**Supplementary Figure 1. Microscopy observation of representative paraffin sections of lotus petiole and peduncle in transverse and vertical directions.**

**
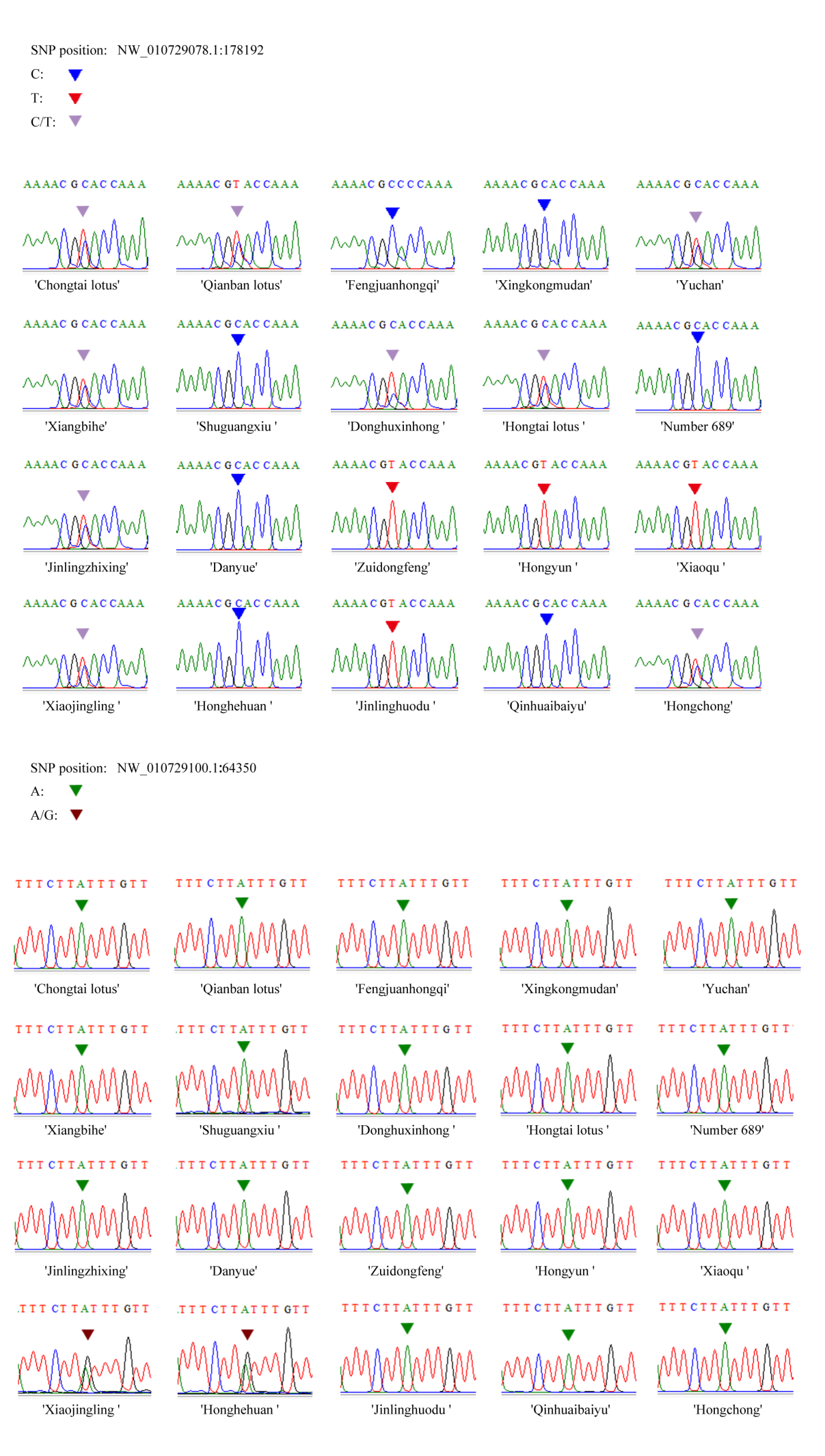
**

**Supplementary Figure 2.** **Genotypes of the 20 re-sequenced lotus germplasms detected by two representative single nucleotide polymorphisms (SNPs) out of the 31 SNPs used in accuracy validation.** SNPs were validated in the 20 re-sequenced lotus germplasms using PCR and Sanger sequencing in triplicate.

**
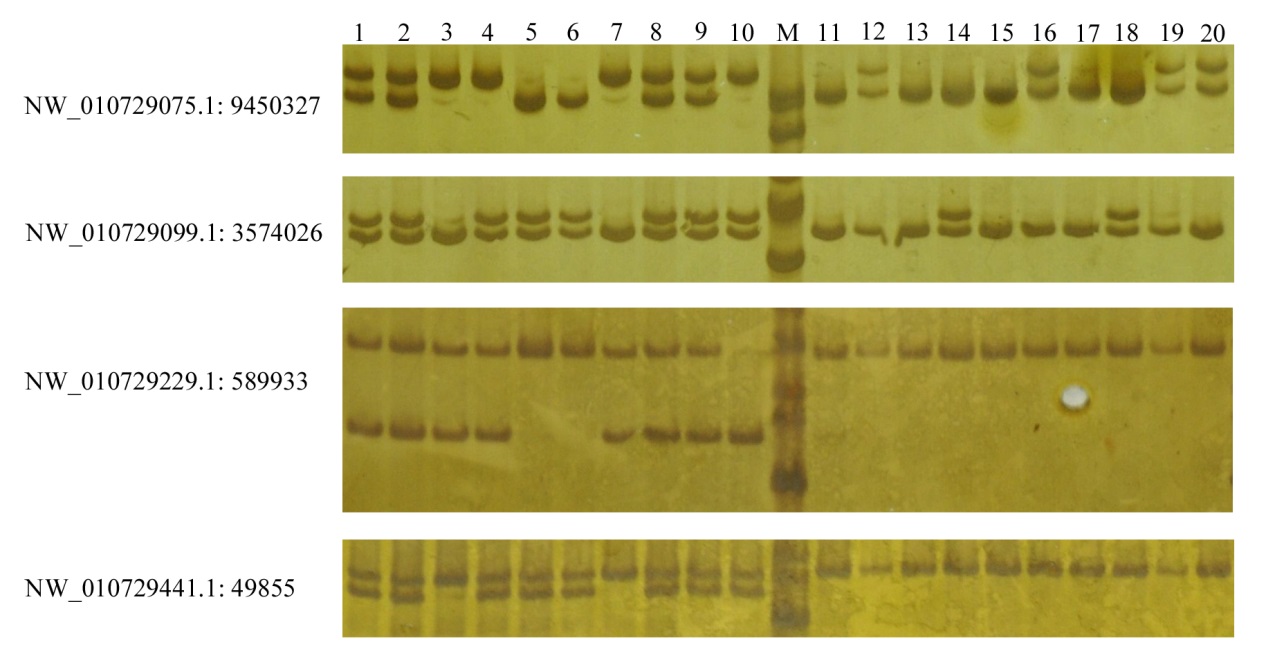
**

**Supplementary Figure 3.** **Polymorphisms of the 20 re-sequenced lotus germplasms detected by four representative InDels among the 30 InDels used in accuracy validation.** Indels were validated in the 20 re-sequenced lotus germplasms using PCR and polyacrylamide gel electrophoresis. 1: Chongtai lotus; 2: Qianban lotus; 3: Fengjuanhongqi; 4: Xingkongmudan; 5: Yuchan; 6: Xiangbihe; 7: Shuguangxiu; 8: Donghuxinhong; 9: Hongtai lotus; 10: Number 689; 11: Jinlingzhixing; 12: Danyue; 13: Zuidongfeng; 14: Hongyun; 15: Xiaoqu; 16: Xiaojingling; 17: Honghehuan; 18: Jinlinghuodu; 19: Qinhuaibaiyu; and 20: Hongchong.


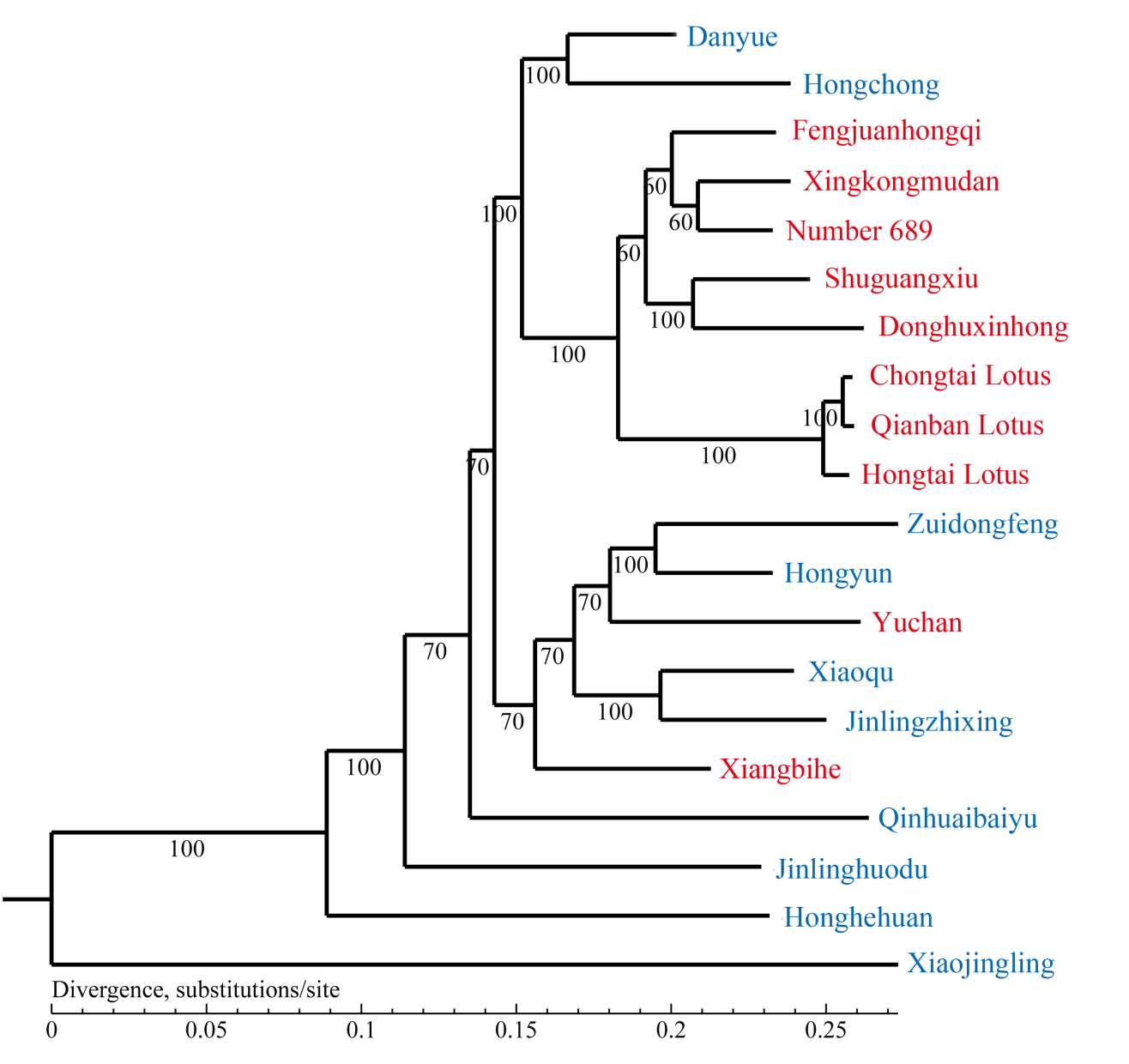


**Supplementary Figure 4. The neighbor-joining phylogenetic tree of the 20 re-sequenced lotus germplasms based on whole-genome SNP data.** Large architecture lotuses and small architecture lotuses are colored in red and blue, respectively.
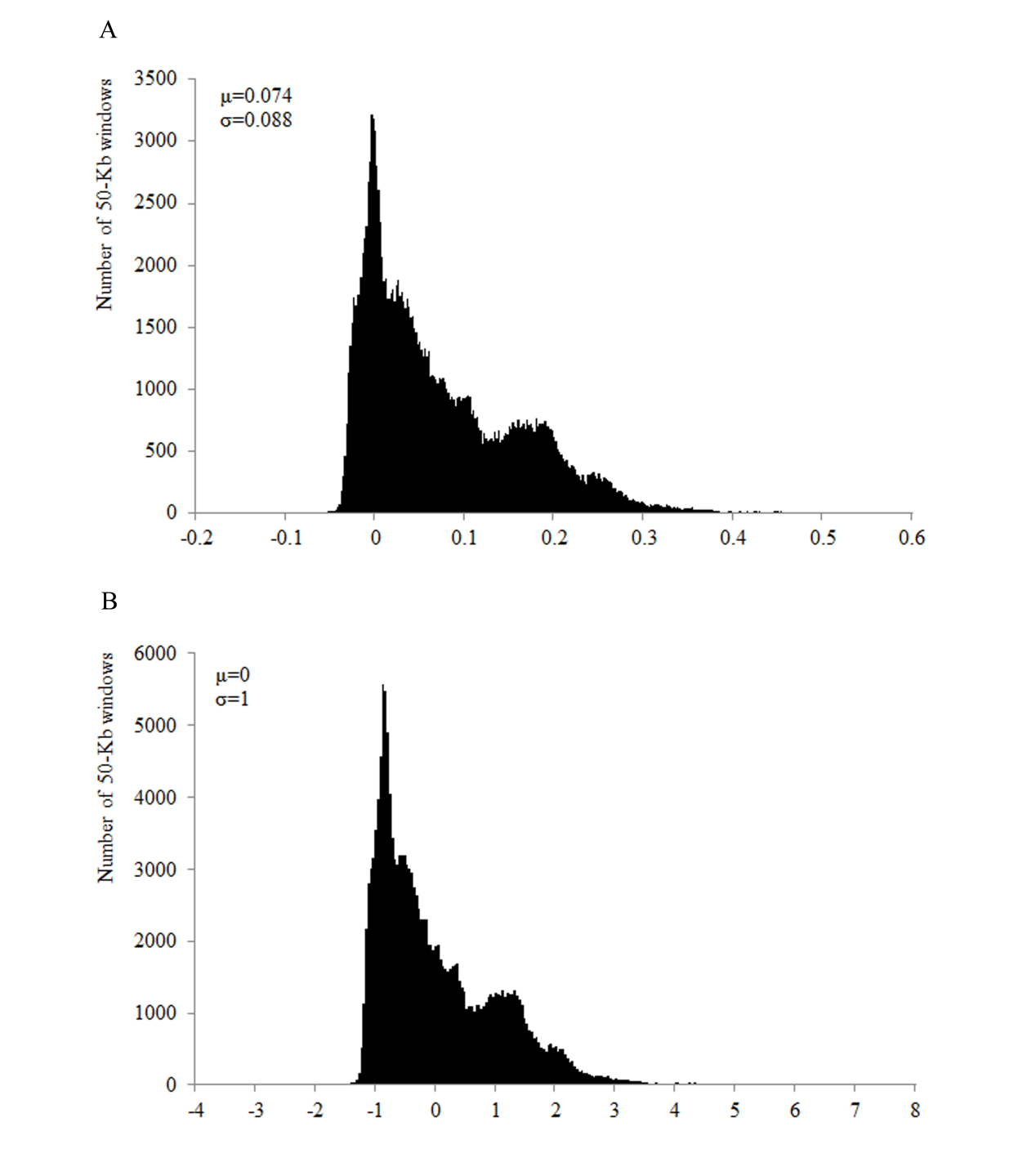


**Supplementary Figure 5.** Distribution of fixation index (F_ST_) values (A) and Z-transformed fixation index (ZF_ST_) values (B) calculated in 50-Kb windows with 5-Kb steps (σ, standard deviation; μ, mean).


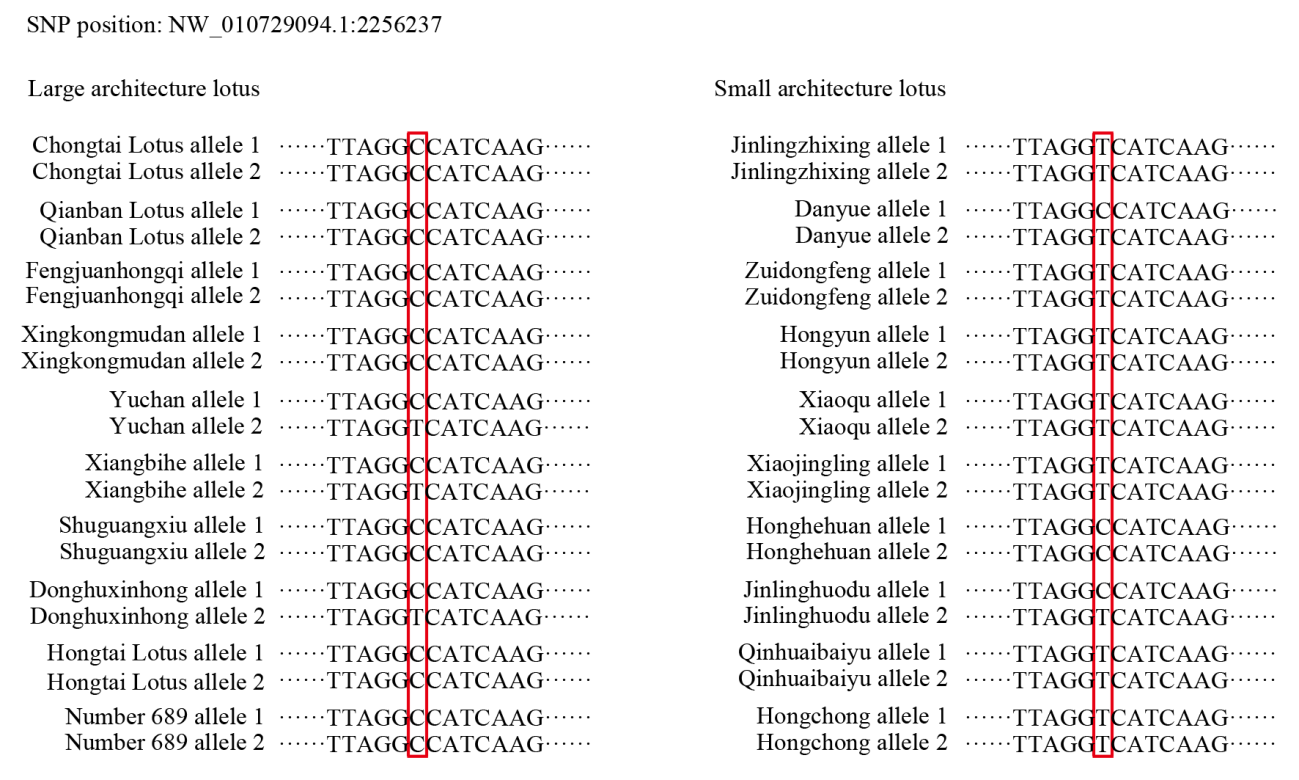


**Supplementary Figure 6.** Diagram of variant with distinct allelic differences between the lotus LA and SA groups by taking SNP located on scaffold NW_010729094.1:2256237 for example. Firstly, the genotype frequencies of the reference genotype (C) and alteration genotype (T) were separately counted in the LA and SA groups. Chi-square test was then applied to analyze the differences in genotype frequency distribution between the two groups. Third, SNP with corrected P value < 0.01 after Benjamini–Hochberg false discovery rate correction was considered significantly differentiated between the LA and SA groups.


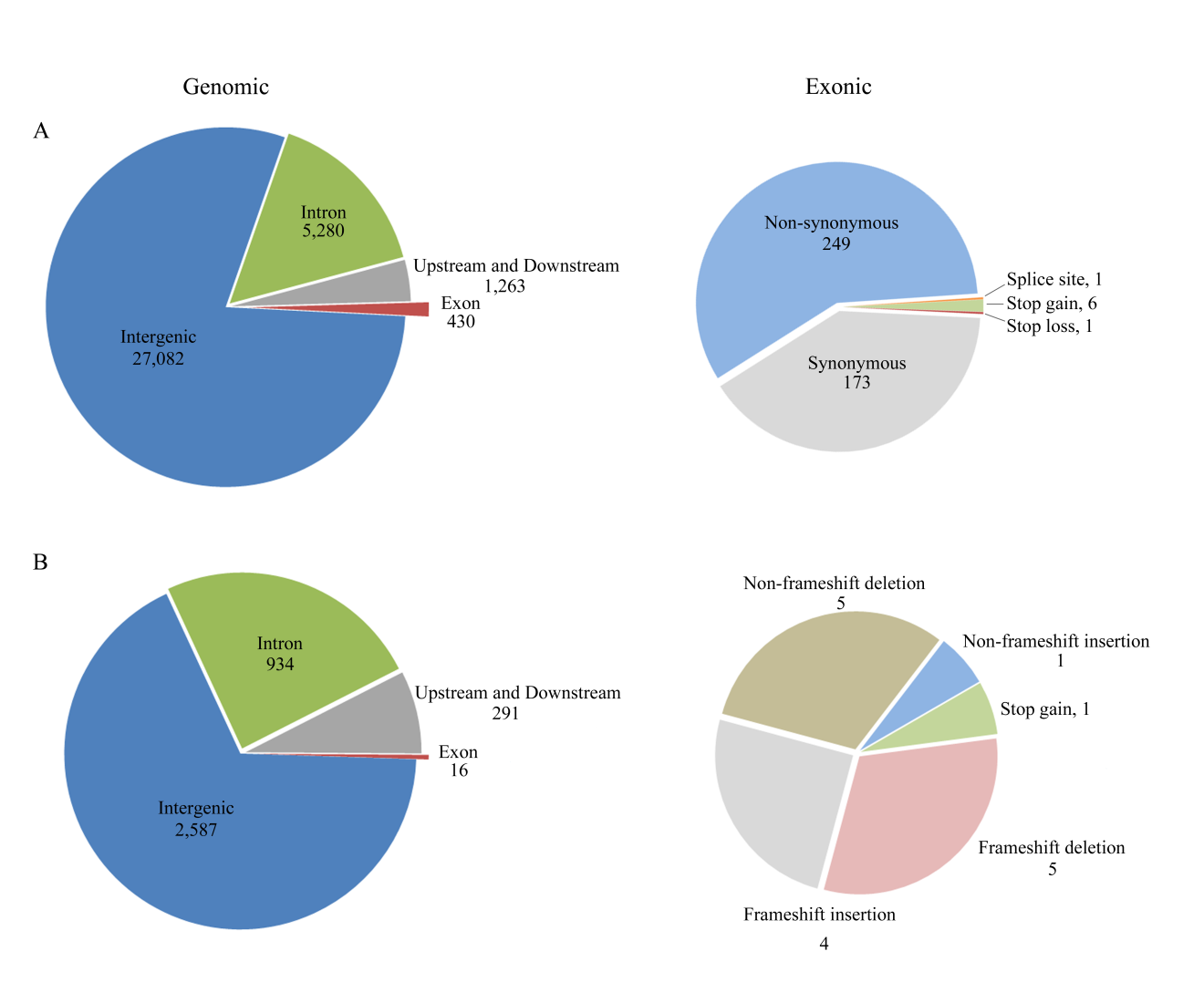


**Supplementary Figure 7.** Functional annotation of SNPs (A) and InDels (B) which showed distinct allelic distributions between the lotus LA and SA groups.


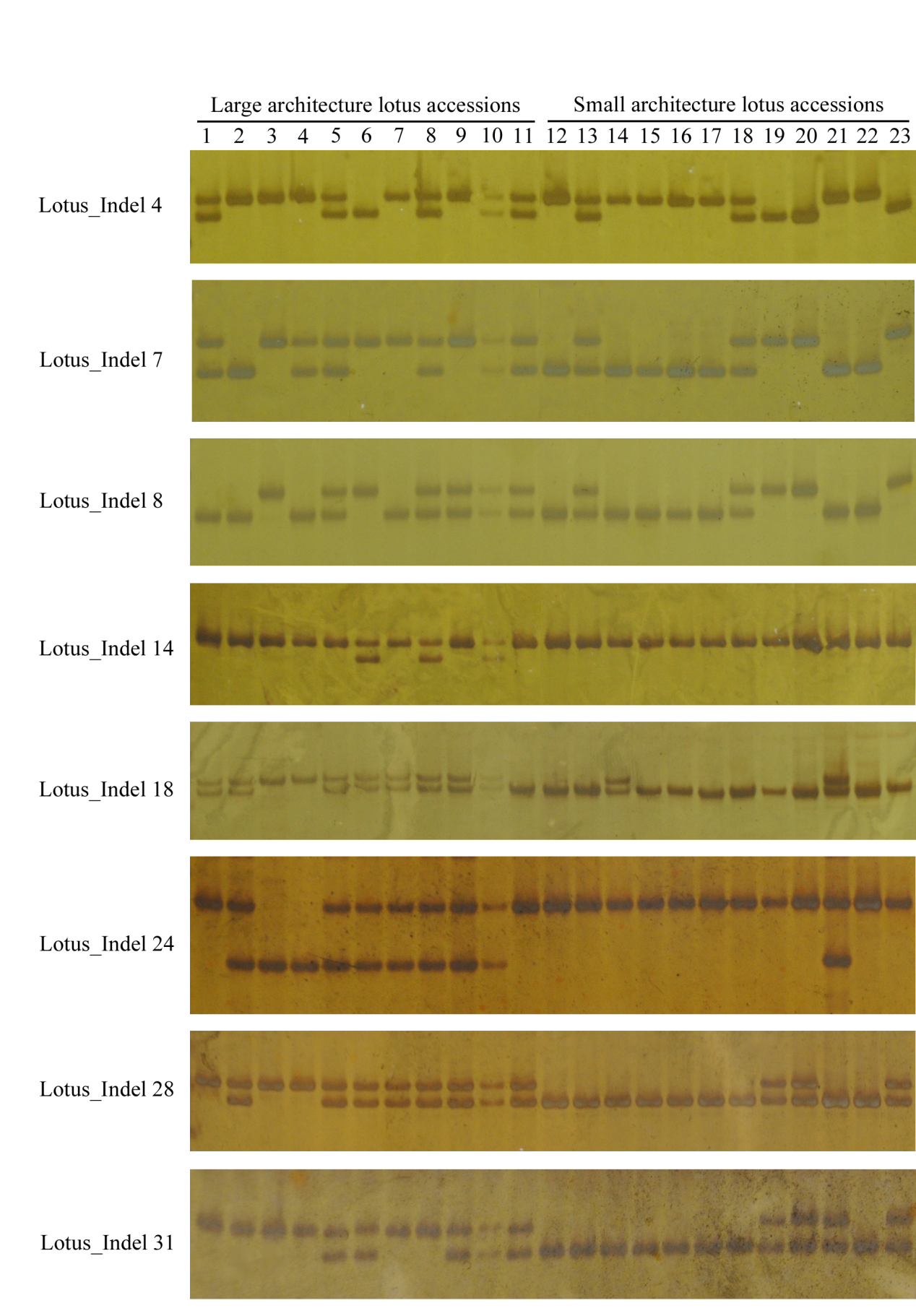


**Supplementary Figure 8.** **Polymorphisms of 23 lotus germplasms detected by eight InDel markers.** 1: Mohe; 2: Benyue; 3: Echenghonglian; 4: Yanermei; 5: Dansajin; 6: Xingkongmudan; 7: Changbanxiaotaohong; 8: Puzheheihonghe; 9: Yizhangyulian; 10: Shaoxinghonglian; 11: Guoqinghong; 12: Jiguang; 13: Jinzhuluoyupan; 14: Dafeng; 15: Lvfanghanzhu; 16: Yanzhilu; 17: Shuimeiren; 18: Ruixue; 19: Xiezhuahong; 20: Yimengqingsi; 21: Qinhuairenjia; 22: Manao; and 23: Xiyanghong. 1–11 are LA lotus germplasms, and 12–23 are SA lotus germplasms.
